# Supplementary figures and images for: Genome-Wide Super-Enhancer-Based Analysis: Identification of Prognostic Genes in Oral Squamous Cell Carcinoma
Source: Int J Mol Sci. 2022 Aug 15;23(16):9154. doi: 10.3390/ijms23169154 (PMC9409227; doi:10.3390/ijms23169154)

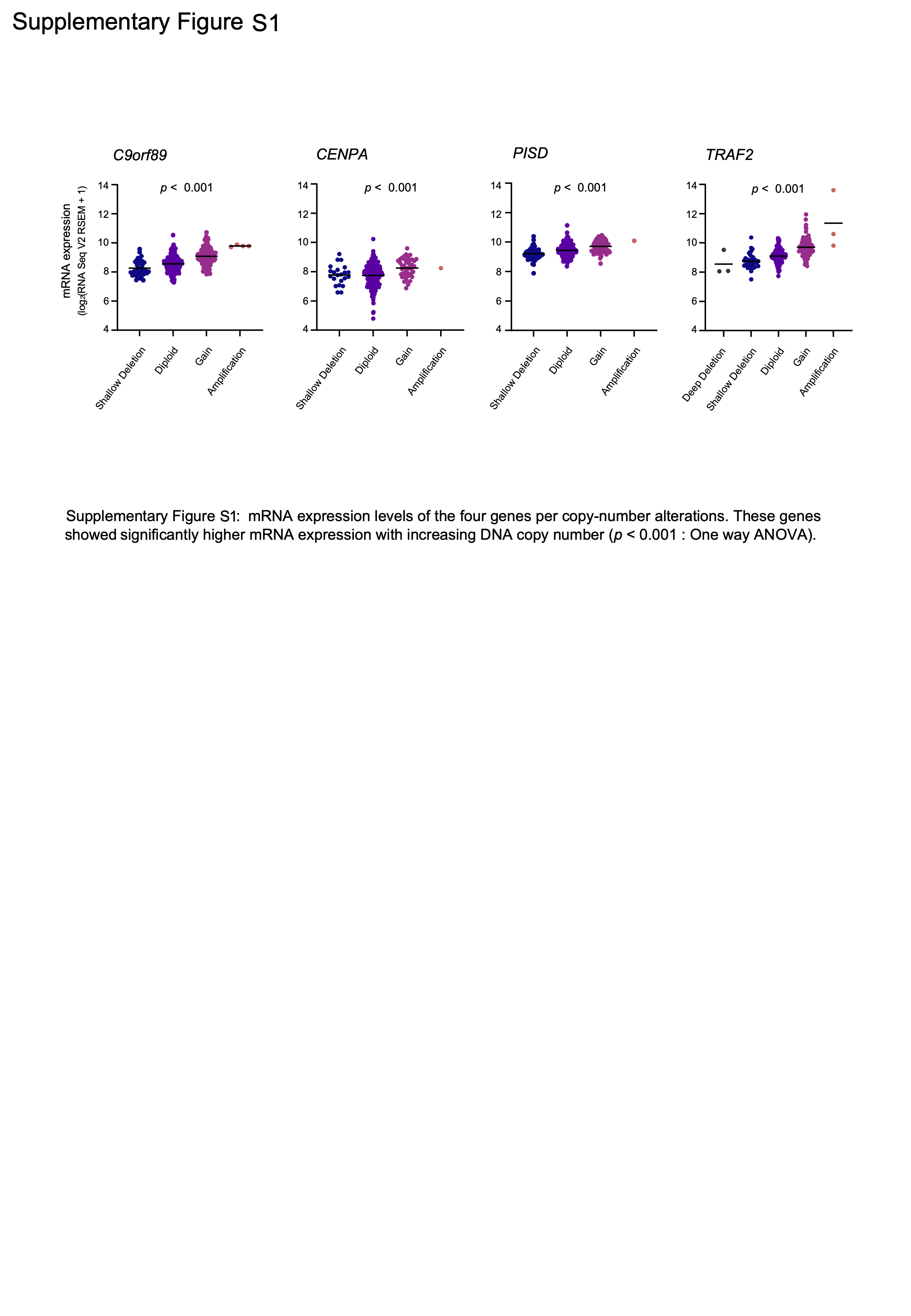

Supplement: Supplementary file 1 [file ijms-23-09154-s001.zip › 20220720 Supplementary Figure S1 Genome-wide super-enhancer-based analysis in OSCC.tiff]

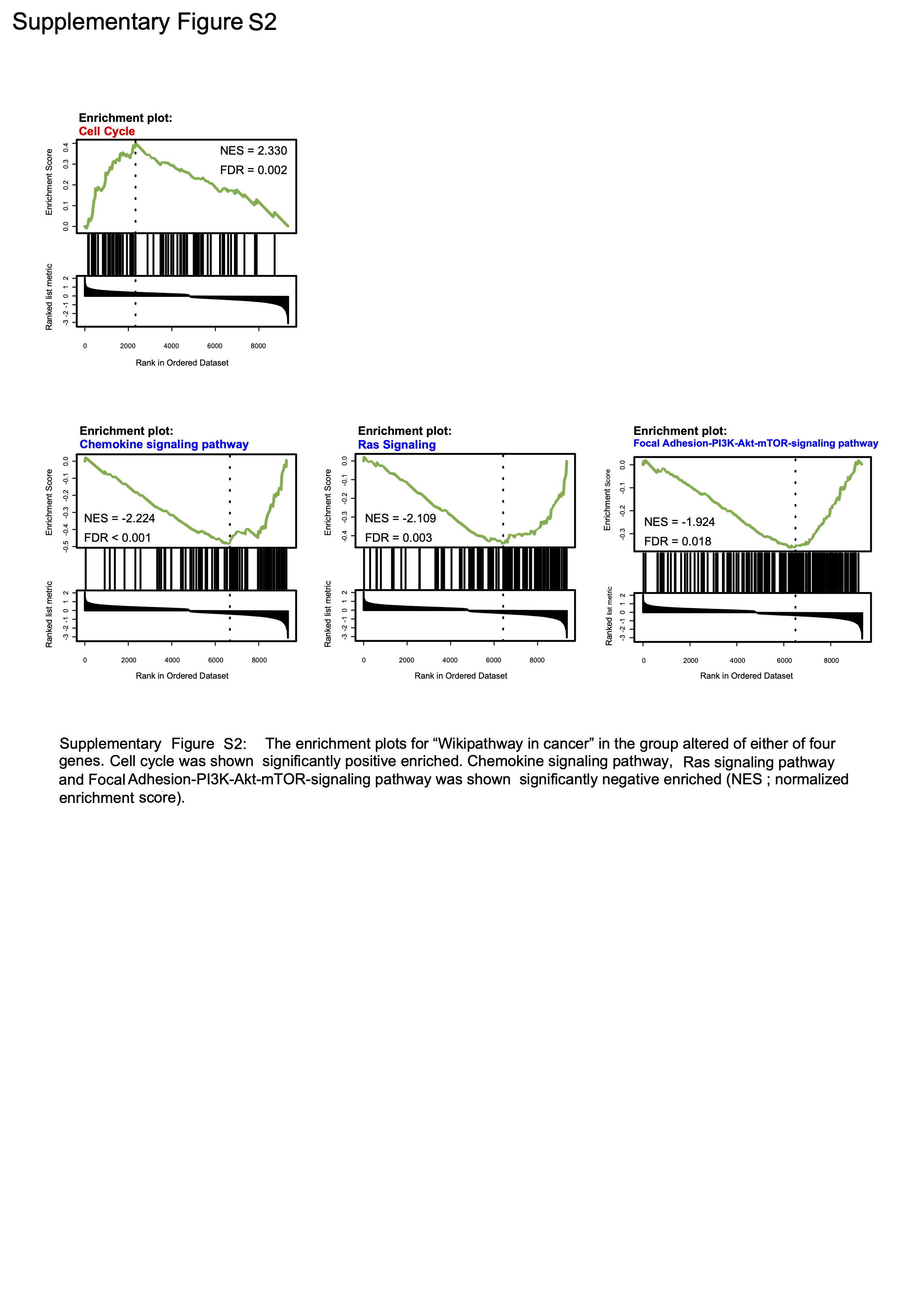

Supplement: Supplementary file 1 [file ijms-23-09154-s001.zip › 20220720 Supplementary Figure S2 Genome-wide super-enhancer-based analysis in OSCC.tiff]
